# A descriptive exploratory study of how admissions caused by medication-related harm are documented within inpatients’ medical records

**DOI:** 10.1186/1472-6963-14-257

**Published:** 2014-06-16

**Authors:** Matthew Reynolds, Mary Hickson, Ann Jacklin, Bryony Dean Franklin

**Affiliations:** 1 Centre for Medication Safety and Service Quality, Imperial College Healthcare NHS Trust and UCL School of Pharmacy, Pharmacy Department, Ground Floor, Charing Cross Hospital, Fulham Palace Road, London W6 8RF, UK; 2 Imperial College London, London SW7 2AZ, UK; 3 Imperial College Healthcare NHS Trust, Charing Cross Hospital, Fulham Palace Road, London W6 8RF, UK

**Keywords:** Hospital admissions, UK, Medical record, Adverse drug reactions, Medication errors, Adherence

## Abstract

**Background:**

Adverse drug reactions, poor patient adherence and errors, here collectively referred to as medication-related harm (MRH), cause around 2.7-8.0% of UK hospital admissions. Communication gaps between successive healthcare providers exist, but little is known about how MRH is recorded in inpatients’ medical records. We describe the presence and quality of MRH documentation for patients admitted to a London teaching hospital due to MRH. Additionally, the international classification of disease 10th revision (ICD-10) codes attributed to confirmed MRH-related admissions were studied to explore appropriateness of their use to identify these patients.

**Methods:**

Clinical pharmacists working on an admissions ward in a UK hospital identified patients admitted due to suspected MRH. Six different data sources in each patient’s medical record, including the discharge summary, were subsequently examined for MRH-related information. Each data source was examined for statements describing the MRH: symptom and diagnosis, identification of the causative agent, and a statement of the action taken or considered. Statements were categorised as ‘explicit’ if unambiguous or ‘implicit’ if open to interpretation. ICD-10 codes attributed to confirmed MRH cases were recorded.

**Results:**

Eighty-four patients were identified over 141 data collection days; 75 met our inclusion criteria. MRH documentation was generally present (855 of 1307 statements were identified; 65%), and usually explicit (705 of 855; 82%). The causative agent had the lowest proportion of explicit statements (139 of 201 statements were explicit; 69%). For two (3%) discharged patients, the causal agent was documented in their paper medical record but not on the discharge summary. Of 64 patients with a confirmed MRH diagnosis at discharge, only six (9%) had a MRH-related ICD-10 code.

**Conclusions:**

Availability of information in the paper medical record needs improving and communication of MRH-related information could be enhanced by using explicit statements and documenting reasons for changing medications. ICD-10 codes underestimate the true occurrence of MRH.

## Background

There are several ways in which use of medicines can lead to harm. First, even correctly prescribed and correctly used medicines can harm through side-effects or unanticipated allergic reactions, ranging from minor to potentially fatal [1]. Second, errors may occur at any step between prescribing and administering a drug: prescribing errors, dispensing errors, monitoring errors and administration errors [2,3] all have potential to cause harm which may be sufficient to warrant hospitalisation [2]. Finally, harm may arise through incorrect use, or non-use, by the patient. In this paper, we use the term medication-related harm (MRH) to encompass harm due to any or all of these three types of harm: adverse drug reactions (ADRs), medication errors, and poor adherence [4]. Other studies focus on adverse drug events; these generally exclude harm due to poor adherence.

While no UK studies have examined the prevalence of hospital admissions caused by MRH encompassing all three types of harm, there are various studies of the separate components. For example, using medical record review, ADRs and adherence problems [4-6] and ADRs exclusively [7,8] have been shown to contribute to 2.7-8.0% of admissions to UK hospitals [4-8]. These figures are comparable to a recent international systematic review [9] which concluded that 5.8% of admissions are caused by ADRs. The estimated cost of ADRs to the National Health Service (NHS) in England is £466 m annually [7]. Harm due to poor adherence, and harm due to medication errors have also been identified as contributing to hospital admissions both in the UK and internationally [4,10].

Patients’ care pathways are complex and multiple care providers are often involved, even during a single hospital stay. With each care decision there is a risk that information may not be passed onto the next care provider in sufficient quality to ensure continuity of decision making and full understanding of previous decisions; such failures in communication have been identified as contributing to medication errors [3,11]. The failure to properly communicate patient information in general, and medication information in particular, has been identified in a number of different healthcare settings [3,11-14], despite guidance on how to document medication changes [11,15]. To ensure continuity of care, it is particularly important to have effective communication between hospital prescribers and community-based General Practitioners (GPs) and community pharmacists who are often responsible for continuing medication started, or changed, in hospital. Within the UK, it has been suggested that incomplete discharge communication, including incomplete medication information, can lead to preventable readmissions [12]. Re-prescribing of medication discontinued or changed in response to MRH can also be responsible for patient re-admission [16,17], although few UK data are available. It is not currently known how MRH information is documented in the UK hospital setting, and whether the nature of MRH documentation contributes to communication gaps between hospital and community settings.

In this exploratory study in one hospital, our aim was to examine how MRH contributing to hospital admission was documented in patients’ medical records at admission, at transfer between hospital wards, and at discharge. Specifically we report on 1) which aspects of MRH were recorded within patients’ medical records and where; 2) what information relating to the MRH and the causal medicines was communicated back to the GP via the hospital discharge communication; 3) how the quantity and quality of information changed throughout the patient’s admission, and 4) which International Classification of Diseases (ICD-10) codes were attributed to MRH-related admissions.

## Methods

### Setting

The study used prospective review of inpatients’ medical records in a large London teaching hospital. Multidisciplinary medical records were mainly paper-based, as is typical at present in the UK, and included various proformas for different aspects of care. For patients admitted via the accident and emergency department (A&E), the triaging nurse completed a brief one-page triage proforma. Subsequently, a multi-page A&E clerking proforma which included an in-depth initial assessment of the initial admission was completed by the admitting A&E doctor. The chronological handwritten case notes and treatment plans provided a diary of patients’ ongoing care. Inpatient prescribing was paper-based, using preformatted drug charts typical within the UK; [18] electronic discharge summaries (Electronic Discharge Communication; EDC) were computer-generated using locally developed discharge prescribing software. Users of the EDC system were prompted to enter information in specific sections which were then printed for dispensing of discharge medication. The EDC comprised a discharge prescription, synopsis of the inpatient admission and recommendations for future management; it was sent to primary care providers after discharge.

Initial patient identification took place on a 26-bed adult acute medical admissions ward. A clinical pharmacist (“admissions pharmacist”) and often a pharmacy technician were present on the ward between 8 am and 6.30 pm every weekday and between 8 am and 1 pm most weekends. The pharmacist attended the multi-disciplinary post-take ward round (PTWR) at 8 am daily, during which the duty medical team reviewed all patients admitted during the previous 24 hours. Diagnoses, decisions and management plans were recorded on a one-page PTWR proforma. The pharmacist and pharmacy technician also performed medication reconciliation on admission, reviewed each patient’s medication, and resolved any problems identified.

The study was registered within the relevant NHS trust as a service evaluation in line with local guidance and did not require ethics approval.

### Data collection

We collected data for patients admitted between 10 October 2011 and 6 June 2012, inclusive of those admitted on weekends and public holidays.

Patients whose admissions were considered to have been caused by MRH were identified by the admissions pharmacists during the PTWR following discussion with the multidisciplinary team; this was an implicit clinical judgement. For each of these patients, we asked the pharmacist to record brief details of the suspected causative medicines, the corresponding MRH, and whether the MRH was considered to be caused by an ADR, an error, or an adherence problem. The pharmacist then evaluated the relationship between the MRH and the hospital admission using Hallas’ criteria [19].

**Figure 1:**
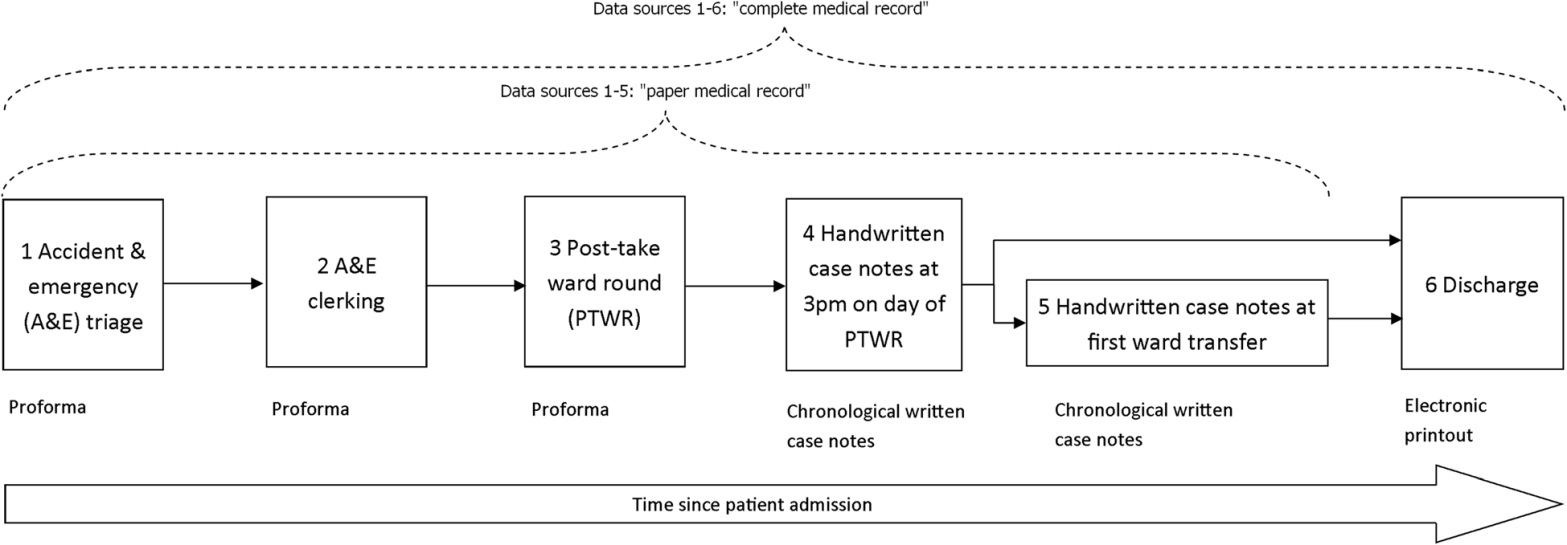
Overview of the six data sources showing which sections of the medical record were examined.

The investigator (MR) examined the pertinent sections of the medical record of each included patient as soon as possible after admission, and shortly after transfer or discharge from the admissions ward. In total, six sections of the complete medical record were examined; each section corresponded to one of six chronological time-points during a typical admission. At each of these six time-points the relevant section of the medical record (“data source”) was examined (Figure 1) and the presence and quality of each of four different aspects of MRH (MRH statements, Table 1) was recorded. All days of the patient’s admission were eligible for data collection.Five of the six chronological points corresponded to transfers between hospital departments or providers of care; we also studied entries in the handwritten case notes up to and including 3 pm on the day of the PTWR (data source 4, Figure 1). According to local guidelines, this is the latest time by which patients reviewed on that morning’s PTWR should have received medication reconciliation and thus any additional information relating to MRH would be documented by that time. Data collected from source 4 enabled the content of the handwritten case notes of patients discharged home from the admissions ward to be compared with case notes of patients transferred to other wards. Information on the inpatient drug chart was considered part of data sources 4 and 5.

**Table 1:** Medication-related harm (MRH) statements

**Statement number**	**Statement relating to**	**Example**
1.	The MRH symptom	“fall”
2.	The MRH diagnosis	“drug-induced hypotension”
3.	Identification of the causative drug	“hypotension caused by anti-hypertensives”
4.	The action already taken or planned in relation to the causative agent	“bisoprolol stopped”

This was an exploratory study therefore no formal sample-size calculation was performed. The admissions pharmacists review approximately 20 patients daily during the PTWR; based on the existing literature [4-9] we therefore expected around one or two admissions each day to be caused by MRH. After discussion with our admissions pharmacists, about five days of data collection per week seemed feasible, and thus a cohort of about 225 patients with MRH over the 30 week course of the study was expected, assuming 5 days data collection per week.

### Inclusion and exclusion criteria

Patients were included in our analysis if the following criteria were met:

• MRH was judged shortly after admission to be the ‘dominant’ cause or ‘partly contributing’ to the admission [19],

• the patient was admitted via A&E and then transferred to the admissions ward,

• the identifying pharmacist’s original summary was detailed enough to permit the investigator to identify the patient,•data were subsequently collected from one or more data sources (Figure 1).

Patients were excluded if they were admitted due to malicious poisoning or deliberate overdose. Daily data collection from the paper medical record was suspended for any patients moved to a critical care area to avoid disrupting care and to minimise unnecessary visits to these areas as per infection control guidelines; the medical record was instead reviewed after patient de-escalation. Data collection for a given patient ceased if their admission was subsequently confirmed to be unrelated to MRH, however data from previous data sources recorded prior to a non-MRH diagnosis being established were included in the analysis. The EDCs for patients subsequently diagnosed with a non-MRH problem were only examined for a statement of symptoms.

### Classification of MRH statements

Four different statements of MRH (Table 1) were sought from each of the six data sources with the medical record (Figure 1). These four statements were based on professional guidelines [15] as well as local policy in relation to what should be recorded in patients’ medical records.

The presence or absence of each of these four types of statement in each data source was established. Identified statements were then defined as either ‘explicit’, if the statement was judged to be unambiguous, or ‘implicit’ for those open to interpretation. If multiple statements were identified, the most specific was recorded.

### Data analysis

Descriptive statistics were used to present the presence of implicit and explicit statements of MRH in each of the six data sources.

To assess inter-rater reliability of the researcher’s interpretations of MRH statements, a senior pharmacist external to the project independently categorised 180 MRH statements from nine randomly selected patients’ complete medical records as explicit, implicit, or absent. These classifications were compared with the investigator’s using a weighted kappa statistic with quadratic weighting [20].

### ICD-10 codes

All unique ICD-10 codes attributed to each patient with a documented confirmed or suspected diagnosis of MRH were examined for codes related to ADRs, unintentional poor adherence, or substances used in error (codes Y40-59, X40-X49, T36-T50 and 58 other individual MRH-related codes, Additional file 1)

## Results

### Admissions due to MRH

**Figure 2:**
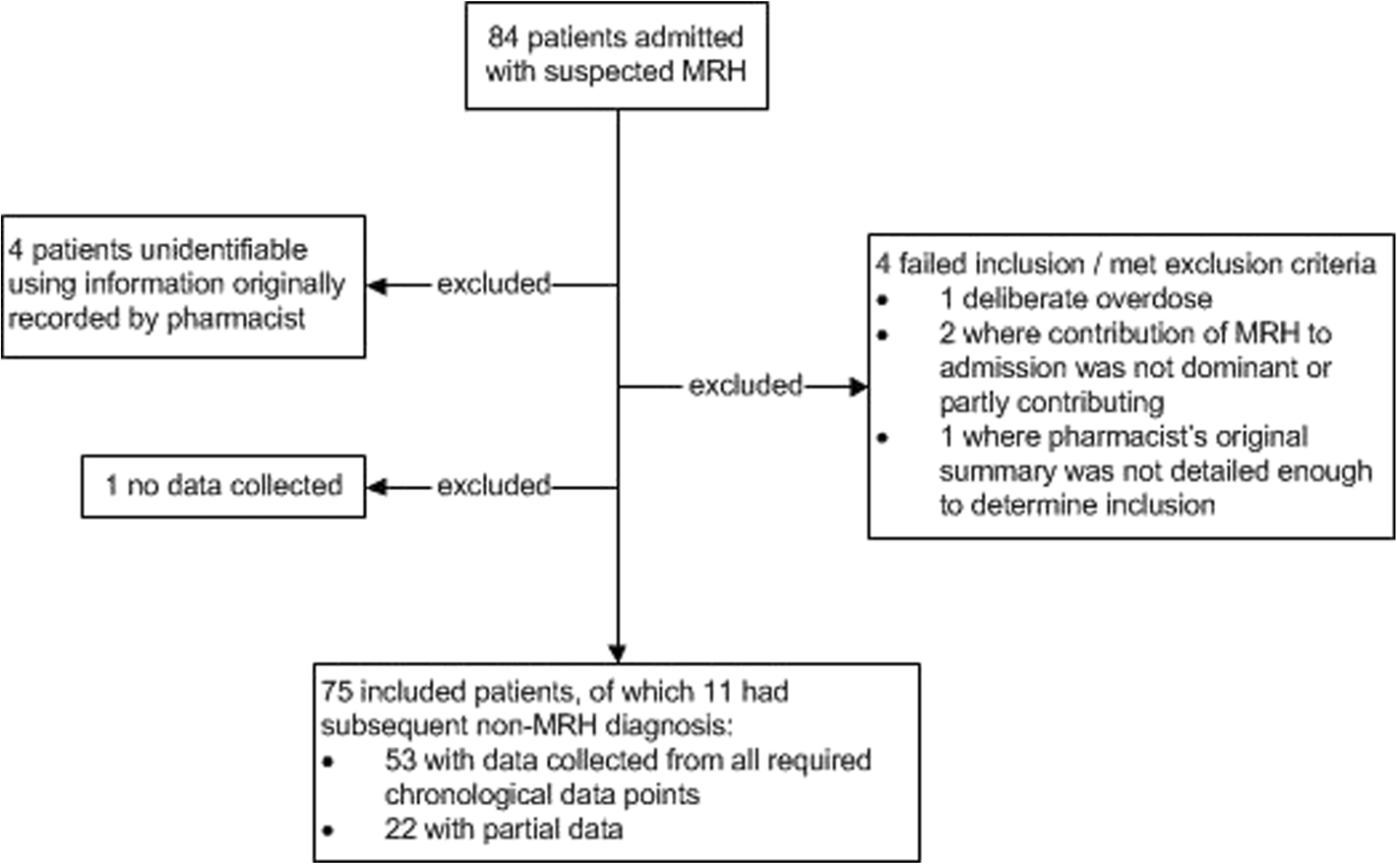
Patient inclusion flowchart (MRH = Medication-related harm).

A total of eight admission pharmacists collected data on 141 (59%) of 240 days between 10 October 2011 and 6 June 2012; pharmacists’ routine workload prevented data collection on the remaining days. Of the 1,237 patients reviewed by admission pharmacists, 84 (6.8%) were suspected shortly after admission as being admitted due to MRH. Of these, nine were subsequently excluded (Figure 2); 75 (6.1% of all patients reviewed) were therefore included in the analysis.

### MRH types and drugs implicated in admissions

The types of MRH suspected as causing admission are categorised in Table 2; 95 different drugs were implicated in the 64 MRH-related admissions. Some admissions were related to multiple drugs.

**Table 2:** Types of suspected medication-related harm admissions and drugs implicated

**Drug group**	**Adverse drug reactions (ADR)**	**Adherence**	**Error**	**ADR & Error**	**Adherence & Error**	**Total**
Diuretics	16	1				17
Anti-epileptics	2	8			1	11
Drugs used in diabetes	2	4	1			7
Beta blockers	4	2				6
Anti-platelets	5					5
Anticoagulants	4	1				5
Opioid analgesics	4	1				5
Drugs affecting the renin-angiotensin system	3	1	1			5
Calcium channel blockers	3	1				4
Anti-infectives	3			1		4
Anti-depressants	3					3
Positive inotropes	2	1				3
Other groups implicated once	15	3	2			20
Total	66	23	4	1	1	95
Total patients	45 (70%)	15 (23%)	2 (3%)	1 (2%)	1 (2%)	64 (100%)

Groups based on British National Formulary [1] categories.

Most admissions were caused by ADRs or adherence problems. Overall, 64/75 (85%) patients initially suspected as being admitted due to MRH subsequently had a diagnosis of MRH confirmed and recorded within their medical record. The remaining 11/75 (15%) patients were subsequently diagnosed with a non-MRH-related problem by the medical team; these patients’ EDCs were therefore examined for statements of symptoms only.

### Extraction of MRH information from the complete medical record

Patients had either five or six possible sources of data examined depending on whether they changed wards as an inpatient. This resulted in a total of 409 data sources for 75 patients. Complete data were collected for 53 (71%) patients (n = 291 data sources). Medical records were missing single data sources for four of the 53 patients: all the available data were collected and they were therefore classified as complete. For the remaining 22 (29%) patients, 44 (37%) of 118 possible data sources were examined. Overall, data were collected from 335 (82%) of the 409 potential chronological data sources.

For 16 patients, data could not be collected from the first four data sources as the paper medical record was sent to central storage before data collection was completed. The paper medical records of a further six patients were also sent to central storage before the case notes at transfer could be examined.

### Documentation of medication related harm within the complete medical record

In total 855/1307 (65%) of all possible statements were present, either implicitly or explicitly. The presence of MRH-related information in each of the data sources varied considerably, Table 3.

**Table 3:** Overview of statement presence in each of the six data sources

**Data source**	**No. of statements judged explicit (% of total possible statements)**	**No. of statements judged implicit (% of total possible statements)**	**Total/possible (% of total possible statements)**
1. Accident & Emergency (A&E) Triage form	88 (37%)	23 (10%)	111/236 (47%)
2. A&E Clerking form	146 (63%)	21 (9%)	167/232 (72%)
3. Post-take ward round form	144 (50%)	34 (12%)	178/228 (78%)
4. Handwritten case notes at 3 pm	95 (42%)	17 (7%)	112/228 (49%)
5. Handwritten case notes at transfer	32 (28%)	8 (7%)	40/116 (35%)
6. Electronic Discharge Communication	200 (75%)	47 (18%)	247/267 (93%)
Total	705	150	855/1307 (65%)

Symptoms were documented well at A&E triage (55 statements in 59 A&E Triage documents examined, 93%). An increase in documentation of diagnosis, identification of the causative agent, and action or plan then occurred at A&E clerking, see Additional file 2. For example, the suspected causative agent was stated in 17/59 (29%) of A&E Triage data sources and in 40/58 (69%) of A&E Clerking data sources.

When all data sources within the paper medical record (sources 1–5) were considered as a whole, the major symptom was mentioned at least once within the entire record in 58 (98%) of 59 cases, the diagnosis in 55 (93%), the causative agent in 54 (92%), and a statement of the action/plan in 52 (88%). Explicit statements of the symptom were found in 58 (98%) of all cases, of diagnosis in 50 (85%), of causative agent in 47 (80%), and of action/plan in 49 (83%) of all cases.

### MRH information availability on the discharge communication

The EDC was the most complete individual data source overall, with 247 (93%) of 267 possible statements present, Table 3. Of these 247 statements, 200 (81%) were explicit and 47 (19%) implicit. The symptoms of MRH were mentioned in 68 (91%) of 75 cases, and explicitly in 65 (96%) of those. The greatest ambiguity was found in the MRH diagnosis statements: 59 (92%) of 64 statements were present, with only 38 (64%) explicit. The causative agent was stated in 60 (94%) of 64 cases, with 43 (72%) explicit. A statement of the action taken or plan for the causative agent was found in 60 (94%) of 64 possible instances; 54 (90%) were explicit.

In total, information regarding 14 statements was not transferred from the paper medical record to the EDC; these concerned six cases of symptom information, four of diagnosis information, and two each of causative agent and action taken/plan.

Conversely, seven additional statements were found on the EDC which were not found in any of the data sources within the paper medical record; four of these related to the action taken/ future plan for the causative agent. The full breakdown of results is presented in Additional file 2.

In some cases, although relevant statements were present on the EDC and thus included in our data, they were located in a section not intended for clinical communication, such as the ‘details of information booklets and other information given with medicines’ section and so potentially would be missed by recipients of this documentation.

### Inter-rater reliability

The weighted kappa statistic for inter-rater reliability of whether statements were explicit, implicit or absent was 0.5, corresponding to ‘fair agreement’ [20].

### Coding of admissions

The 64 patients with a confirmed diagnosis of MRH somewhere in their complete medical record had 475 ICD-10 codes assigned (median 7, range 2–15). One patient admitted with adherence problems had an MRH-related primary diagnosis (assigned ICD-10 code E16.0, drug-induced hypoglycaemia without coma) and one secondary diagnosis (Y42.3, Drugs, medicaments and biological substances causing adverse effects in therapeutic use, Insulin and oral hypoglycaemic drugs). Three patients admitted due to ADRs were assigned two MRH codes in the secondary diagnosis field, and two further patients admitted due to ADRs were assigned one relevant code. A total of six (9%) of 64 patients therefore had one or more MRH-related ICD-10 codes assigned to their admission; five of the six had explicit statements relating to the MRH diagnosis on their EDC, Table 4. Of the 58 patients not attributed a MRH-related ICD-10 code, 33 (57%) had an explicit diagnosis on their EDC, 21 (36%) were implicit, and 4 (7%) had none.

**Table 4:** Electronic Discharge Communication statements, with associated International Classification of Diseases (ICD) 10 codes

**Excerpt no.**	**Statement**	**ICD-10 code**
1.	*“main condition: drug induced neutropaenia”*	Y41.0
2.	“*the diagnosis was lactic acidosis, secondary to EtOH [ethanol] excess and metformin”*	Y49.2 and Y51.3
3.	*“main condition: medication induced delirium”*	Y49.2 and Y51.3
4.	*“main condition: lithium toxicity”*	X49.9
5.	*“Digoxin (change reason, stopped due to toxicity)”*	X44.9 and T46.0

## Discussion

We found 64/1237 (5.2%) of admissions to be caused by MRH. In general, this MRH was fairly well documented in all sources examined within the complete medical record. However, failure to transfer information from inpatients’ medical records to discharge summaries was identified, and there was no one place within the paper medical record where complete MRH information was consistently found. Finally, less than one in ten patients with a confirmed MRH-related diagnosis had this reflected in their ICD-10 codes.

### Interpretation and implications for practice

The A&E triage data source contained a symptom statement in 93% cases. A substantially higher percentage of A&E clerking documents included statements of diagnosis, identification of the causative agent, and action or plan than A&E triage.

The one-page PTWR form should capture concise, specific information about a patient’s diagnosis and management plan. Documentation of all potential MRH statements was found in at least 65% of the PTWR data sources. The percentage of implicit statements within this source was relatively high (23% of all cases). However, the PTWR doctors’ time–pressure [21] may be reflected in the use of the single-word or very short statements identified, perhaps assuming the next reader will link together the information on the PTWR form with that mentioned elsewhere within the patient’s medical record [22].

Of interest was the contribution the contents of the drug chart made to increasing overall documentation in the 3 pm data source. This suggests that the drug chart is not only used as a record of prescribed and administered drugs, but is sometimes used to communicate clinical information which may not be found elsewhere. As electronic prescribing systems become more widespread, it will be important to consider whether these systems can also be used to document this additional contextual information.

In total, information regarding 14 statements was not transferred from the paper medical record to the EDC; conversely, seven additional statements were found on the EDC and not in the paper medical record. The presence of additional statements on the EDC may reflect new information discovered during the patient’s stay. The data collection method for this study focussed primarily on the first two or three days after admission, and at discharge. Information regarding a patient’s condition which was established after first ward-transfer and documented in the paper medical notes before discharge was not collected. However, most of the information presented at discharge was identified during the first two or three days of patients’ hospital stays.

One aspect of MRH where a degradation of information seemed to occur was illustrated by the decrease in percentage availability of symptom and diagnosis statements between paper medical record and the EDC: there were six instances of symptom information not being transferred to EDCs, and four instances of diagnosis information not being transferred. This failure to transfer information may be a reflection of the way the information is spread throughout the inpatient record making comprehensive transfer of information difficult. Patients’ medical records are often large and comprise many pages. To provide an accurate discharge summary, the discharging doctor must condense and communicate information dispersed throughout the entire paper medical record: they must be able to identify the most recent and accurate data entries on which to base their discharge summary. It may be unreasonable to expect a discharging doctor to review every patient’s entire medical record and extract all relevant information. Electronic records may allow storage of clinically important information in easier-to-navigate patient chronologies, which would allow ready access to the most up-to-date information. However, given current variation in extent and approach to electronic health record system use within the UK, more work is likely to be needed to realise these potential benefits [23].

Four or five different data sources were examined in the paper medical record compared with one at discharge; therefore poor documentation within individual sources in the paper medical record does not necessarily translate into poor cumulative availability when patients’ paper medical records are considered as a whole.

Overall, our findings suggest that while key information is generally transferred onto the EDC, important information is sometimes lost. As failure to pass on information has been identified as a patient care problem [3,11-14] there may be potential to avoid injury at minimal cost to the NHS by encouraging and facilitating better communication of the information which already exists in patients’ medical records.

Regarding the structure of the EDC specifically, there were instances when the statement relating the causative agent to the symptom or diagnosis was in an inappropriate section of the EDC. The person who made the entry was not always stated, but some entries were specifically attributable to pharmacists. For instance, relevant information was sometimes found in the “details of information booklets and other information given with medicines” section or the “additional notes” section where pharmacists can add additional comments. The pharmacist may add valuable medicine-related information to the EDC but the next reader may not be alerted to this information because it is printed in an unexpected section of the EDC. Future EDC systems must allow both doctors and pharmacists to make entries in a section of the EDC dedicated to medication changes, and to enable easy documentation of MRH.

The role of patient adherence as a contributing factor to admission was sometimes poorly communicated on the EDC; in one instance there was no indication that a patient admitted following a collapse had mistakenly taken their anti-hypertensive medicines twice. Adherence problems commonly fall to primary care providers to manage; it is therefore crucial that this be communicated to GPs and to community pharmacists wherever possible. However, as the patient is discharged with a copy of the EDC, the discharging doctor may be reluctant to apportion them blame and possibly cause offence, or wary of making statements based on potentially inaccurate assumptions in the medical records. Discharging doctors are unlikely to have admitted the patient, and may not have built a sufficient relationship with the patient to document culpability.

ICDs related to MRH were rarely used. Although we studied a small sample of patients from one organisation, the lack of MRH-related coding was notable. Both health care planning and research are underpinned by good quality evidence based on accurate data. Since coders use patients’ complete medical records as the primary source of information for coding, the accuracy of coding is likely to reflect the clarity of that medical record. Although MRH information was present in most cases, it was often in a format which needed interpretation, and was spread throughout the entire record. The EDC often provides a more interpretable (81% explicit statements) and accessible summary, and use of unambiguous statements (e.g. drug induced neutropenia) may facilitate assignment of MRH-related ICD codes.

### Implications for research

Investigation into the reasons for the ambiguousness of some information on the PTWR form data source may enable improvement of the PTWR form, or contribute to the restructuring of the PTWR. Future research should also explore how GPs, community pharmacists, patients and other recipients interpret MRH-related information on patients’ discharge communications and how this information is acted upon. The reasons for failing to document adherence problems should also be explored, as should the reasons why doctors use implicit or short statements within the medical record, and whether use of explicit or implicit statements is related to the probability that the admission was MRH-related.

Any work reporting a rate of MRH-related admissions calculated only using routinely collected ICD-10 codes is likely to underestimate the true occurrence. Exploring the reasons why ICD-10 codes are not used for MRH-related admissions should be a next step.

### Comparison with existing literature

The 5.2% (64 of 1,237) of patients admitted and diagnosed with MRH is in line with previous UK studies [4-6] using similar data collection methods, but focussing only on admissions caused by ADRs and poor adherence, which report 5.3-8.0%, and with international figures [9,24]. We also included admissions due to errors but these were responsible for only five admissions. We also found the causative agents to be similar to those reported elsewhere [25].

The ICD codes attributed to each hospital admission have previously been used to identify ADR-related admissions [26,27], while others suggest that using ICD codes underestimates their true occurrence [28,29].

In our study a total of 6 (0.5%; 95% confidence interval 0.1-0.9%) of 1237 admissions were found to have one or more MRH-related ICD-10 codes. Wu et al. [26] found 557,978 of 59,718,694 (0.9%) of admissions over a ten year period to have an ADR-related ICD-10 code. When Wu et al.’s inclusion criteria are applied to the present sample, only 3 (0.2%; 95% confidence interval 0.0-0.5%) of our 1237 admissions had an ICD-10 code from the selection of ICD codes used by Wu et al. It is not clear why the proportion of admissions coded as being due to ADRs was lower in our study, but may be due to our sample being much smaller and from a more specific admission pathway in just one hospital.

### Strengths and limitations

As well as being the first study to explore these issues, a particular strength of our study is that MRH-related admissions were detected during the PTWR, when each patient is examined by a multi-disciplinary team with great experience in detecting causes of admission, thus the diagnosis of MRH reflects the opinion of a team, rather than a single individual.

Limitations include the fact that the contents of the patient’s handwritten case notes were only examined up to the time of first ward transfer (typically two or three days after admission). As patients in our sample stayed for up to 65 days, it is unsurprising that extra information was found on the discharge summary when compared with those data sources examined in the first few days of admission. Furthermore, we were not able to capture information from every data source for every patient, reducing the completeness of the data set. Data collection took place between October and June and so any seasonal variation may not be fully represented.

The inter-rater reliability testing performed for categorising the presence of MRH-related information showed only ‘fair’ agreement, and our sample was relatively small. We did not explore whether agreement was affected by the profession of the assessor. Finally, while we recorded whether or not MRH was predominantly caused by error we did not assess the preventability of other types of MRH detected.

## Conclusion

MRH was documented fairly well throughout the paper medical record and at discharge. However, in line with previous work, communication gaps were found. Instances where relevant information within the paper medical record was omitted from the EDC were discovered. Complete MRH information was not consistently found in any single source within the medical record, which may contribute to gaps in communication. Closing these gaps may reduce the burden of MRH on the NHS and individual patients. The limitations of using ICD codes to identify MRH should be clearly understood before drawing inferences from them.

## Abbreviations

A&E: Accident and emergency; ADR: Adverse drug reaction; EDC: Electronic discharge communication; GP: General Practitioner; ICD: International Classification of Diseases; MRH: Medication-related harm; NHS: National Health Service; PTWR: Post-take ward round.

## Competing interests

All authors declare no competing interests.

## Authors’ contributions

MR and BDF conceived the study. MR, BDF and MH participated in the study’s design. MR collected all data. All authors contributed to the interpretation of the results, and to drafting the final manuscript. All authors have read and approved the final manuscript.

## Pre-publication history

The pre-publication history for this paper can be accessed here:

http://www.biomedcentral.com/1472-6963/14/257/prepub

## Supplementary Material

Additional file 1 **International classification of diseases codes covering medication-related harm.** This table shows all included international classification of disease (ICD-10) codes, and is referenced in the text as “Additional file 1”. Click here for file

Additional file 2 **Presence of implicit and explicit statements throughout the complete medical record.** This table shows all results from each data source, and is referenced in the text as “Additional file 2”. Footnotes: *‘Cumulative availability’ was defined as the presence of a statement within the medical notes up and including the data source in question. In cases where explicit and implicit statements are present, only the explicit statement is counted in the numerator. #‘Cumulative opportunity to state’ is the total number of individual patients for whom at least one data source was examined up to and including that data source. Click here for file
