# Supplementary material for: A descriptive exploratory study of how admissions caused by medication-related harm are documented within inpatients’ medical records
Source: BMC Health Serv Res. 2014 Jun 16;14:257. doi: 10.1186/1472-6963-14-257 (PMC4072847; doi:10.1186/1472-6963-14-257)
Supplement: Additional file 1 — International classification of diseases codes covering medication-related harm. This table shows all included international classification of disease (ICD-10) codes, and is referenced in the text as “Additional file 1”. [file 1472-6963-14-257-S1.docx]

| Chapter | Chapter Title | Code | Description | Included by Wu et al. 2010 |
| --- | --- | --- | --- | --- |
| III | Diseases of the blood | D52.1 | Drug-induced folate deficiency anaemia | Yes |
|  |  | D59.0 | Drug induced autoimmune haemolytic anaemia | Yes |
|  |  | D59.2 | Drug induced nonautoimmune haemolytic anaemia | Yes |
|  |  | D61.1 | Drug induced aplastic anaemia | Yes |
| IV | Endocrine, nutritional and metabolic diseases | E06.4 | Drug-induced thyroiditis | Yes |
|  |  | E16.0 | Drug-induced hypoglycaemia without coma | Yes |
|  |  | E23.1 | Drug-induced hypopituitarism | Yes |
|  |  | E24.2 | Drug-induced Cushing’s syndrome | Yes |
|  |  | E27.3 | Drug-induced adrenocortical insufficiency | Yes |
|  |  | E66.1 | Drug-induced obesity | Yes |
| V | Mental and behavioural disorders | F13 | Mental and behavioural disorders due to use of sedatives or hypnotics | Yes |
|  |  | F19 | Mental and behavioural disorders due to multiple drug use and use of other psychoactive substances | Yes |
| VI | Diseases of the nervous system | G21.0 | Malignant neuroleptic syndrome | Yes |
|  |  | G21.1 | Other drug-induced secondary parkinsonism | Yes |
|  |  | G24.0 | Drug-induced dystonia | Yes |
|  |  | G25.1 | Drug-induced tremor | Yes |
|  |  | G25.4 | Drug-induced chorea | Yes |
|  |  | G25.6 | Drug-induced tics | Yes |
|  |  | G44.4 | Drug-induced headache, not elsewhere classified | Yes |
|  |  | G62.0 | Drug-induced polyneuropathy | Yes |
|  |  | G72.0 | Drug-induced myopathy | Yes |
| VII | Diseases of the eye and adnexa | H26.3 | Drug-induced cataract | Yes |
|  |  | H91.0 | Ototoxic hearing loss | Yes |
| IX | Diseases of the circulatory system | I42.7 | Cardiomyopathy due to drugs and other external agents | Yes |
|  |  | I95.2 | Hypotension due to drugs | Yes |
| X | Diseases of the respiratory system | J70.2 | Acute drug-induced interstitial lung disorders | Yes |
|  |  | J70.3 | Chronic drug-induced interstitial lung disorders | Yes |
|  |  | J70.4 | Drug-induced interstitial lung disorders, unspecified | Yes |
| XI | Diseases of the digestive system | K71 | Toxic liver disease | Yes |
| XII | Diseases of the skin and subcutaneous tissue | L23.3 | Allergic contact dermatitis due to drugs in contact with skin | Yes |
|  |  | L24.4 | Irritant contact dermatitis due to drugs in contact with skin | Yes |
|  |  | L25.1 | Unspecified contact dermatitis due to drugs in contact with skin | Yes |
|  |  | L27.0 | Generalized skin eruption due to drugs and medicaments | Yes |
|  |  | L27.1 | Localized skin eruption due to drugs and medicaments | Yes |
|  |  | L51.2 | Toxic epidermal necrolysis [Lyell] | Yes |
|  |  | L56.0 | Drug phototoxic response | Yes |
|  |  | L56.1 | Drug photoallergic response | Yes |
| XIII | Diseases of the musculoskeletal system and connective tissue | M02.2 | Postimmunization arthropathy | Yes |
|  |  | M10.2 | Drug-induced gout | Yes |
|  |  | M32.0 | Drug-induced systemic lupus erythematosus | Yes |
|  |  | M34.2 | Systemic sclerosis induced by drugs and chemicals | Yes |
|  |  | M80.4 | Drug-induced osteoporosis with pathological fracture | Yes |
|  |  | M81.4 | Drug-induced osteoporosis without pathological fracture | Yes |
|  |  | M83.5 | Other drug-induced osteomalacia in adults | Yes |
|  |  | M87.1 | Osteonecrosis due to drugs | Yes |
| XIV | Diseases of the genitourinary system | N14.1 | Nephropathy induced by other drugs, medicaments and biological substances | Yes |
|  |  | N14.2 | Nephropathy induced by unspecified drug, medicament or biological substance | Yes |
| XIX | Injury, poisoning and certain other consequences of external causes | T36-T50 | Poisoning by drugs, medicaments and biological substances | No |
|  |  | T80.5 | Complications following infusion, transfusion and therapeutic injection: anaphylactic shock due to serum | Yes |
|  |  | T80.6 | Complications following infusion, transfusion and therapeutic injection: other serum reactions | Yes |
|  |  | T80.8 | Other complications following infusion, transfusion and therapeutic injection | Yes |
|  |  | T80.9 | Unspecified complication following infusion, transfusion and therapeutic injection | Yes |
|  |  | T88.0 | Infection following immunization | Yes |
|  |  | T88.1 | Infection complications following immunization | Yes |
|  |  | T88.2 | Shock due to anaesthesia | Yes |
|  |  | T88.3 | Malignant hyperthermia due to anaesthesia | Yes |
|  |  | T88.6 | Anaphylactic shock due to adverse effect of correct drug or medicament properly administered | Yes |
|  |  | T88.7 | Unspecified adverse effect of drug or medicament | Yes |
| XX | External causes of morbidity and mortality | X40-X59 | Accidental poisoning by and exposure to noxious substances | No |
|  |  | X44.9 | Accidental poisoning by and exposure to noxious substances - Accidental poisoning by and exposure to other and unspecified drugs, medicaments and biological substances | No |
|  |  | X49.9 | Accidental poisoning by and exposure to noxious substances - Accidental poisoning by and exposure to other and unspecified chemicals and noxious substances | No |
|  |  | Y40-Y59 | Drugs, medicaments and biological substances causing adverse effects in therapeutic use | Yes |
|  |  | Y41.0 | Drugs, medicaments and biological substances causing adverse effects in therapeutic use - Other systemic anti-infectives and antiparasitics - Sulfonamides | Yes |
|  |  | Y42.3 | Drugs, medicaments and biological substances causing adverse effects in therapeutic use - Hormones and their synthetic substitutes and antagonists, not elsewhere classified - Insulin and oral hypoglycaemic [antidiabetic] drugs | Yes |
|  |  | Y46.0 | Drugs, medicaments and biological substances causing adverse effects in therapeutic use - Antiepileptics and antiparkinsonism drugs - Succinimides | Yes |
|  |  | Y49.2 | Drugs, medicaments and biological substances causing adverse effects in therapeutic use - Psychotropic drugs, not elsewhere classified - Other and unspecified antidepressants | Yes |
|  |  | Y51.3 | Drugs, medicaments and biological substances causing adverse effects in therapeutic use - Drugs primarily affecting the autonomic nervous system - Other parasympatholytics [anticholinergics and antimuscarinics] and spasmolytics, not elsewhere classified | Yes |
