# Supplementary material for: A descriptive exploratory study of how admissions caused by medication-related harm are documented within inpatients’ medical records
Source: BMC Health Serv Res. 2014 Jun 16;14:257. doi: 10.1186/1472-6963-14-257 (PMC4072847; doi:10.1186/1472-6963-14-257)
Supplement: Additional file 2 — Presence of implicit and explicit statements throughout the complete medical record. This table shows all results from each data source, and is referenced in the text as “Additional file 2”. Footnotes: *‘Cumulative availability’ was defined as the presence of a statement within the medical notes up and including the data source in question. In cases where explicit and implicit statements are present, only the explicit statement is counted in the numerator. #‘Cumulative opportunity to state’ is the total number of individual patients for whom at least one data source was examined up to and including that data source. [file 1472-6963-14-257-S2.docx]

| Data source | Statement |  | Number of times stated in data source | Patients with valid data source | % stated in data source |  | Explicit statements in data source | |  | Implicit statements in data source | |  | Cumulative availability of explicit statement* | |  | Cumulative availability of implicit statement* | |  | Cumulative availability of any statement* | Cumulative opportunity to state# | % times cumulatively available |
| --- | --- | --- | --- | --- | --- | --- | --- | --- | --- | --- | --- | --- | --- | --- | --- | --- | --- | --- | --- | --- | --- |
| Accident & emergency (A&E) Triage | MRH symptom |  | 55 | 59 | 93% |  | 54 | 92% |  | 1 | 2% |  | 54 | 92% |  | 1 | 2% |  | 55 | 59 | 93% |
|  | MRH diagnosis |  | 29 | 59 | 49% |  | 17 | 29% |  | 12 | 20% |  | 17 | 29% |  | 12 | 20% |  | 29 | 59 | 49% |
|  | Causative agent |  | 17 | 59 | 29% |  | 10 | 17% |  | 7 | 12% |  | 10 | 17% |  | 7 | 12% |  | 17 | 59 | 29% |
|  | Action / Plan |  | 10 | 59 | 17% |  | 7 | 12% |  | 3 | 5% |  | 7 | 12% |  | 3 | 5% |  | 10 | 59 | 17% |
| A&E clerking | MRH symptom |  | 55 | 58 | 95% |  | 52 | 90% |  | 3 | 5% |  | 58 | 98% |  | 0 | 0% |  | 58 | 59 | 98% |
|  | MRH diagnosis |  | 42 | 58 | 72% |  | 36 | 62% |  | 6 | 10% |  | 41 | 69% |  | 9 | 15% |  | 50 | 59 | 85% |
|  | Causative agent |  | 40 | 58 | 69% |  | 30 | 52% |  | 10 | 17% |  | 33 | 56% |  | 9 | 15% |  | 42 | 59 | 71% |
|  | Action / Plan |  | 30 | 58 | 52% |  | 28 | 48% |  | 2 | 3% |  | 29 | 49% |  | 3 | 5% |  | 32 | 59 | 54% |
| Post-take ward round (PTWR) | MRH symptom |  | 52 | 57 | 91% |  | 48 | 84% |  | 4 | 7% |  | 58 | 98% |  | 0 | 0% |  | 58 | 59 | 98% |
|  | MRH diagnosis |  | 46 | 57 | 81% |  | 38 | 67% |  | 8 | 14% |  | 49 | 83% |  | 5 | 8% |  | 54 | 59 | 92% |
|  | Causative agent |  | 43 | 57 | 75% |  | 27 | 47% |  | 16 | 28% |  | 42 | 71% |  | 10 | 17% |  | 52 | 59 | 88% |
|  | Action / Plan |  | 37 | 57 | 65% |  | 31 | 54% |  | 6 | 11% |  | 43 | 73% |  | 4 | 7% |  | 47 | 59 | 80% |
| Hand-written case notes, 3pm | MRH symptom |  | 28 | 57 | 49% |  | 26 | 46% |  | 2 | 4% |  | 58 | 98% |  | 0 | 0% |  | 58 | 59 | 98% |
|  | MRH diagnosis |  | 31 | 57 | 54% |  | 26 | 46% |  | 5 | 9% |  | 50 | 85% |  | 4 | 7% |  | 54 | 59 | 92% |
|  | Causative agent |  | 29 | 57 | 51% |  | 20 | 35% |  | 9 | 16% |  | 44 | 75% |  | 8 | 14% |  | 52 | 59 | 88% |
|  | Action / Plan |  | 24 | 57 | 42% |  | 23 | 40% |  | 1 | 2% |  | 47 | 80% |  | 4 | 7% |  | 51 | 59 | 86% |
| Hand-written case notes at first ward transfer | MRH symptom |  | 10 | 29 | 34% |  | 9 | 31% |  | 1 | 3% |  | 58 | 98% |  | 0 | 0% |  | 58 | 59 | 98% |
|  | MRH diagnosis |  | 11 | 29 | 38% |  | 8 | 28% |  | 3 | 10% |  | 50 | 85% |  | 5 | 8% |  | 55 | 59 | 93% |
|  | Causative agent |  | 10 | 29 | 34% |  | 7 | 24% |  | 3 | 10% |  | 47 | 80% |  | 7 | 12% |  | 54 | 59 | 92% |
|  | Action / Plan |  | 9 | 29 | 31% |  | 8 | 28% |  | 1 | 3% |  | 49 | 83% |  | 3 | 5% |  | 52 | 59 | 88% |
| Discharge | MRH symptom |  | 68 | 75 | 91% |  | 65 | 87% |  | 3 | 4% |  | n/a | n/a |  | n/a | n/a |  | n/a | n/a | n/a |
|  | MRH diagnosis |  | 59 | 64 | 92% |  | 38 | 59% |  | 21 | 33% |  |  |  |  |  |  |  |  |  |  |
|  | Causative agent |  | 60 | 64 | 94% |  | 43 | 67% |  | 17 | 27% |  |  |  |  |  |  |  |  |  |  |
|  | Action / Plan |  | 60 | 64 | 94% |  | 54 | 84% |  | 6 | 9% |  |  |  |  |  |  |  |  |  |  |
